# Consuming an All-Meat Ketogenic Diet for the Long-Term Management of Candida Vulvovaginitis and Vaginal Hidradenitis Suppurativa: A 47-Month Follow-Up Case Report

**DOI:** 10.7759/cureus.30510

**Published:** 2022-10-20

**Authors:** Nadia Yar, Lawrance T Mukona, Kim Nguyen, Linette Nalbandyan, Lorraine Mukona, Guinda St. Fleur, Norman L Lamberty, Kyle Zullo, Adam Le, Alex Van, Brandon Allen

**Affiliations:** 1 College of Medicine, American University of Antigua, Antigua, ATG; 2 Psychiatry, American University of Antigua, Antigua, ATG; 3 Obstetrics and Gynecology, American University of Antigua, Antigua, ATG; 4 Obstetrics and Gynecology, Montclair Medical Center, Montclair, USA; 5 Internal Medicine, Medical University of the Americas, Charlestown, KNA

**Keywords:** candida infection, a case report, classic ketogenic diet, hiradenitis suppurativa, candida vulvovaginitis

## Abstract

This case report describes long-term therapeutic management in a 33-year-old diagnosed with Candida vulvovaginitis and vulvar hidradenitis suppurativa 47 months previously. Candida spp. yeasts are part of many women's normal vaginal microflora, and the development of vulvovaginal candidiasis is typically a result of a disturbance in the patient's microbial ecosystem, which manifests itself by intense pruritus, erythema, swelling, and thick white vaginal discharge. Hidradenitis suppurativa is a chronic auto-inflammatory skin condition that causes painful weeping lesions in areas of dense apocrine glands. Although certain mechanisms underlying the pathogenesis of Hidradenitis Suppurativa (e.g., risk factors include smoking, obesity, and family history) have been investigated, a definitive explanation remains elusive.

Nutritional intervention in the form of an all-meat ketogenic diet may be considered therapy in the management of both diseases, as successfully seen in this case report. The patient refused standard of care with oral fluconazole for Candida vulvovaginitis and surgical removal for Hidradenitis suppurativa, and instead consumed a zero-carbohydrate all-meat ketogenic diet mostly of beef with strict adherence to the diet for 43 days in which symptoms ceased.

## Introduction

Candida spp. usually live as commensal organisms that seldom produce illness; however, it is the most prevalent fungal pathogen of humans when it does. As a well-known colonizer of mammals, Candida spp. predominantly resides in the skin, gastrointestinal and genitourinary tracts of humans. Thus, Candida spp. infections are mostly confined to the skin or mucous membranes and can be an irritating recurrence in some women as Candida vulvovaginitis. Despite the high efficacy of azole derivative medications, little research has been done regarding nutrition that may or may not impact mucocutaneous Candida prognoses.

Another cutaneous inflammatory process, hidradenitis suppurativa is a chronic, auto-inflammatory skin condition that affects approximately 0.1% of the population of the United States [1]. In the absence of curative treatment despite recurring discomfort, shame, and scarring, hidradenitis suppurativa severely deters the quality of life in affected patients. As a result, Hidradenitis patients frequently experiment with lifestyle modifications such as diet in an effort to alleviate symptoms.

The ketogenic diet is a high-fat, low-carbohydrate dietary therapy. Ketogenesis is activated with gluconeogenesis during fasting, starvation, or the consumption of low carbohydrates. This paper explores a unique case in the therapeutic success of consuming an all-meat ketogenic diet for the long-term management of Candida vulvovaginitis and vaginal hidradenitis suppurativa.

## Case presentation

The patient, a 29-year-old white American female with no significant past medical history including any gynecological history, the patient has never had PCOS, fibroids, menorrhagia, dysmenorrhea, dyspareunia, or any other medical/gynecological issues. The patient used no vaginal douches, soaps, scented mixtures of any sort, and owned no pets. This remained consistent in order to prevent changes to her natural vaginal flora. The patient has no significant sexual history and has not had any sexual activity for the last seven years. Prior, the patient's last sexual and only encounter was in 2014 with one partner. The patient presented on July 16, 2018, at HFMC Women’s Health Center, Michigan, US, for a routine gynecological examination. On physical examination, the vaginal canal appeared erythematous with thick, white, curd-like discharge. Additionally, three hyper-pigmented, scarred, minimally raised vulvar plaques with subcutaneous sinus tracts were all leaking fluid. No past medical history or family history of any previous illness was reported. The patient’s height, weight and body mass index (BMI) were 5’8” (172cm), 227 lbs. (102.965 kg), and 34.6 kg/m^2^, respectively. Pulse and blood pressure were within normal limits (64 bpm and 125/76 correspondingly). Laboratory investigation indicated blood chemistry within normal limits. Blood Glucose and WBC Count were within normal limits (88mg/dL and 4.2 K/L, respectively). No imaging studies were recommended for patient. Further items from the patient’s laboratory analysis included: Sodium 136mmol/L, Potassium 3.9mmol/L, Chloride 106mmol/L, Carbon Dioxide 24 mmol/L, Anion Gap 6, Creatinine 0.81 mg/dL, Calcium 9.3 mg/dL, GFR 96 ml/min/1.73m2 (all within normal limits).

**Figure 1:**
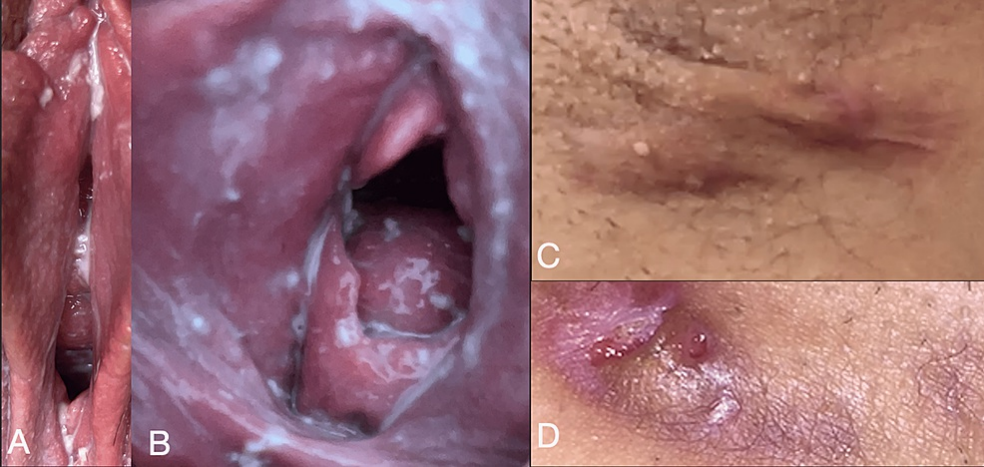
Gross images of the patient’s Candida vulvovaginitis (A, B) and hidradenitis suppurativa (C, D)

The initial clinical impression was Candida vulvovaginitis and vulvar abscesses as shown in Figures 1A-1D. Gynecological cytopathology (reported July 23, 2018) confirmed the presence of fungal organisms morphologically consistent with Candida albicans. Surgical pathology determined that the lesions included free hair embedded in granulomatous inflammation, fibrosis, and ruptured follicular cysts, consistent with hidradenitis suppurativa.

The patient was given a referral to the dermatology department for surgical removal of the cutaneous lesions and prescribed fluconazole for her Candida vulvovaginitis. The patient was instructed to take one 150mg fluconazole tablet on Day 1, Day 4, and Day 7, then weekly for six months. Azole derivatives, a single daily or weekly dose of 150 mg are sufficient to treat mucocutaneous candidiasis. Despite insistence from healthcare providers, the patient refused the standard of care, nor did she schedule an appointment with a dermatologist. Instead, she replaced the directed treatment with the implementation of a zero-carbohydrate all-meat ketogenic diet, consisting predominantly of grass-fed beef ribeye steak and ground beef patties. Her regimen consisted of 70% of total daily calories from fat (approximately 2,120 kCal) and 30% protein (approximately 890 kCal).

The patient strictly adhered to the diet, using Trividia Acetoacetic Acid reagent strips for urinalysis to monitor her urine acetoacetic acid value (mmol), as well as the keto-mojo Glucose and Ketone kit to monitor plasma levels. She then calculated her glucose/ketone index to target therapeutic ketosis (glucose/ketone index 1.0-3.0). The patient reported that she required approximately nine days to reach trace, followed by small and moderate levels of acetoacetic acid in her urine. It took the patient 17 days to enter the predicted zone of therapeutic ketosis (glucose/ketone index 1.0-3.0). The patient reported that 43 days following the initiation of an all-meat ketogenic diet program, all symptoms ceased. In addition to a full ketogenic diet, the patient did not use any vaginal solutions or soaps after or prior to the infection. The patient consistently used antibacterial, non-scented soaps and avoided any douches or vaginal cleansers in order to avoid any changes in microbiology of natural flora. She continued her diet and on follow-up on January 17, 2019, no signs of vulvovaginitis or hidradenitis suppurativa were reported or appeared on physical examination. No further symptomatology appeared since the patient’s adherence to the diet in further in person follow-up visits on February 26, 2021, January 3, 2022, March 17, 2022, or July 1, 2022.

## Discussion

Medically, ketogenic diets are not the preferred pharmacological preference for candida or HS. Following oral administration, antifungal azole derivatives undergo hepatic biotransformation, culminating in a plasma half-life of 30 hours. Therefore, a single daily or weekly dose of 150 mg is sufficient to treat mucocutaneous candidiasis.

Ketogenesis is activated in concert with gluconeogenesis during fasting, starvation, or minimal carbohydrate intake [2]. During gluconeogenesis, glucagon activation of the cAMP cascade in the liver inhibits glycolysis, therefore limiting pyruvate production from carbohydrates [2]. Pyruvate that is created from lactate or alanine is converted to oxaloacetate by the enzyme pyruvate carboxylase, which is further activated by acetyl-CoA. The oxaloacetate is converted to malate for gluconeogenesis, but due to the low level of oxaloacetate, the acetyl-CoA is directed to ketogenesis rather than used for energy metabolism in Kreb’s cycle [2]. High ATP concentration produced during fat metabolism inhibits the TCA cycle at the isocitrate dehydrogenase step and also inhibits the electron transport chain due to an increase in the mitochondrial membrane potential [2]. The resulting increase in the NADH/NAD+ ratio favors the reduction of oxaloacetate to malate (in order to reverse the ratio and regenerate NAD+), which exits the mitochondrion for gluconeogenesis, instead of consumption in the tricarboxylic acid cycle. Ultimately, acetyl-CoA produced by β-oxidation of fatty acids is converted to ketone bodies.

Ketogenic diets were first popularized in the early 20th century for their efficacy in the management of refractory epilepsy. Since then, it has gained recognition as a concurrent or complementary therapeutic strategy for the management of many cancers and chronic inflammatory diseases [3]. Ketone body metabolism provides normal cells with a metabolic advantage over fungal cells due to increased change in Gibb’s Free Energy of ATP hydrolysis from approximately −56kJ/mole to −59kJ/mole [4]. Normally, the body’s cells (macrophages, neutrophils) phagocytose C. albicans, and oxidative killing is the first line of host defense, illustrated by the increased risk of C. albicans infection in individuals with neutropenia or defects in oxidative enzymes NADPH oxidase or myeloperoxidase [5]. Thus, a more energized arsenal for phagocytes as a result of ketosis improves the elimination of Candida spp. overgrowth. Restricted Ketogenic diets are also anti-inflammatory and can therefore minimize chronic inflammation [4]. Both energized cells of the immune system and the alleviation of chronic inflammation may explain the alleviation of the patient’s symptoms to the ketogenic diet. Here, we demonstrate the beneficial effect of ketogenic diets in Candida vulvovaginitis.

## Conclusions

Despite current literature that informs clinicians to utilize antifungal azole derivatives in the treatment of mucocutaneous Candida spp. infection and surgical removal for the management of vulvar hidradenitis suppurativa, this case study describes long-term successful management of both with a zero-carbohydrate nutritional strategy without the use of medication or invasive surgery. Possible mechanisms by which an all-meat ketogenic diet might reduce Candida spp. growth and eliminate symptoms of vulvar Hidradenitis are discussed in the remainder of this paper. An all-meat ketogenic diet was successfully utilized to treat Candida vulvovaginitis and vulvar hidradenitis suppurativa. Further studies will be needed to test this hypothesis in other patients diagnosed with Candida vulvovaginitis and vulvar hidradenitis suppurativa.
